# Oxymetazoline Hydrochloride Combined With Mometasone Nasal Spray for Persistent Nasal Congestion (Pilot Study)

**DOI:** 10.1097/WOX.0b013e31820f8fae

**Published:** 2011-03-15

**Authors:** Efren L Rael, John Ramey, Richard F Lockey

**Affiliations:** 1 Department of Medicine, Pulmonary, Allergy and Critical Care Medicine, Penn State Milton S. Hershey Medical Center, Hershey, Pennsylvania; 2 National Allergy, Asthma, and Urticaria Centers of Charleston, Charleston, South Carolina; 3 University of South Florida, Department of Allergy/Immunology, James A. Haley Veterans' Administration Hospital, Tampa, Florida

## To the Editor

Nasal glucocorticosteroids (NGCS) are considered first-line therapy for allergic rhinitis and are effective therapy for nonallergic rhinitis, especially vasomotor rhinitis and nonallergic rhinitis with eosinophilia syndrome [1-4]. Nasal congestion can persist despite maximum treatment with NGCS. Although oral and topical antihistamines are effective in relieving this symptom, [5,6] neither oral nor topical antihistamines are as effective in relieving this symptom compared with NGCS [7,8]. Oxymetazoline (OXY) is a decongestant and the addition of oxymetazoline to a NGCS should add a considerable decongestant benefit. However, its use is recommended only for 3-14 days because of the risk of rhinitis medicamentosa (RM), increased nasal congestion caused by prolonged use of such drugs [9].

NGCS are helpful in reducing the amount of rebound congestion in patients with perennial allergic rhinitis with RM. They also decrease nasal mucosal edema, recruitment of neutrophils and mononuclear cells, cytokine production, and late-phase nasal mediators. They may offer a protective benefit from the development of RM. Oxymetazoline may also decrease turbinate enlargement, thereby permitting better nasal distribution of NGCS.

Data are limited about efficacy and safety of OXY used with NGCS for nasal congestion (NC) not adequately responding to recommended doses of NGCS.

## Methods

Patients had at least a 1-year history of perennial allergic or nonallergic rhinitis (including vasomotor rhinitis and nonallergic rhinitis with eosinophilia syndrome) documented by skin testing in the medical record before study enrollment. Those receiving allergen immunotherapy were on a stable maintenance regimen for at least 30 days before the first study visit and remained on the same dose during the study. Institutional review board approval and informed consent were obtained before initiation of the study.

Patients completed twice daily diary cards pertaining to their nasal symptoms: 0 (not noticeable); 1 (mild symptoms, noticeable but not bothersome); 2 (moderate symptoms, noticeable and disturbing some of the time); 3 (severe symptoms, very disturbing some of the time and/or disturbing most of the time). All had a nasal congestion score of 1.5 or greater at the screening visit (day 7) and an average nasal congestion score of 1.5 or greater at the baseline visit (day 1). The average nasal congestion score was calculated from during the last 3 days before baseline and the morning of the baseline visit (i.e., total of 7 scores) [10].

**Figure 1:**
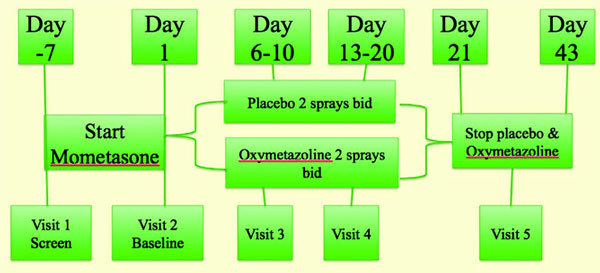
**Study design and treatment arms**.

A 20-day randomized double blind, placebo controlled pilot study of patients ≥18 years with a minimum 1-year history of moderate to severe NC that failed to respond to NGCS were stratified to mometasone nasal spray (MNS) 100 *μ*g every night or nightly and OXY 0.05% 2 sprays per nostril (s/n) 2 times daily versus MNS 100 *μ*g every night or nightly plus saline placebo (PL) 2 s/n 2 times daily (Figure 1).

Patients completed diaries for NC and quality of life (QOL) and underwent intranasal examination for nasal and turbinate changes and rhinorrhea. Patients continued on NGCS after study termination.

## Results

**Figure 2:**
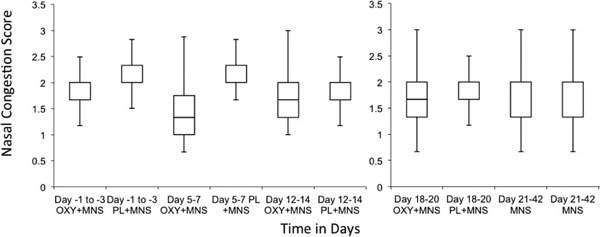
**Nasal congestion score versus time OXY + MNS versus PL + MNS**.

Twenty-three patients met inclusion criteria. The MNS plus OXY cohort versus MNS plus PL achieved an averaged NC score of 1.68 versus 2.06 (NS), respectively (Figure 2). The treatment group reported 22% improvement in average daily NC score on days 5 to 7 (NC score 1.56; 95% confidence interval [CI] 1.23-1.89); 14.5% on days 12 to 14 (NC score 1.71; 95% CI 1.39-2.03); 13.5% on days 18 to 20 (NC score 1.73; 95% CI 1.39-2.07); 6.5% post-treatment days 21 to 42 (NC score 1.87; 95% CI 1.56-2.18) versus the MNS plus PL of 1.4% worsening on days 5 to 7 (NC score 2.16; 95% CI 1.94-2.38); 9.4% improvement on days 12 to 14 (NC score 1.93; 95% CI 1.64-2.22); 6.6% improvement on days 18 to 20 (NC score 1.99; 95% CI 1.61-2.37); and 5.8% improvement post-treatment days 21 to 42 (NC score 1.92; 95% CI 1.55-2.29). With use of Kolmogorov-Smirnov statistical analysis, the *P *value comparing the 2 groups on days 18 to 20 was 0.12. Slightly more nasal erythema occurred with OXY. No RM or other side effects occurred in either group.

The Mini Rhinoconjunctivitis Quality of Life Questionnaire (Mini RQLQ) is stratified into 5 categories: activities, practical problems, nose symptoms, eye symptoms, and other symptoms. Patients rate symptoms from 0 (not troubled) to 6 (extremely troubled).

**Figure 3:**
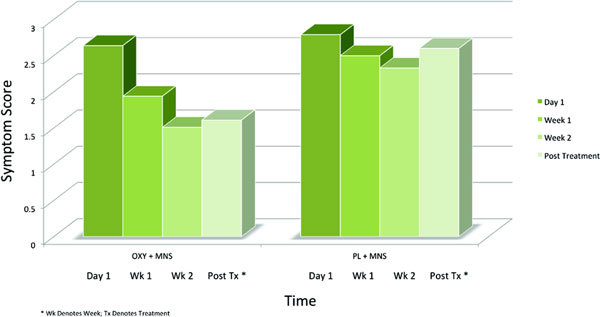
**Mini RQLQ Activity Category: Composite of occupational activities, recreational activities, and sleep symptom scores rated from 0 to 6 (0 = not troubled; 6 = extremely troubled)**.

Among the 5 Mini RQLQ indicators, the MNS plus OXY cohort reported the biggest benefit on activity measures, *P *= 0.04 (Figure 3). The treatment group reported 36% improvement in ability to do regular activities at home/work on days 6 to 10; 45% improvement on days 13 to 20 versus the MNS plus PL 11% improvement on days 6 to 10; 31% improvement on days 13 to 20. Patients in the MNS plus OXY cohort noted improvement in the ability to do recreational activities by 28% on days 6 to 10; 57% improvement on days 13 to 20 versus 15% improvement on days 6 to 10; 22% improvement on days 13 to 20 in the MNS plus PL cohort. Among the activity benefits, sleep improvement was comparatively most consistently beneficial in the MNS plus OXY cohort with 17% improvement on days 6 to 10; 28% on days 13 to 20 versus 4% improvement on days 6 to 10; 2% worsening on days 13 to 20 in the MNS plus PL cohort.

## Conclusions

MNS plus OXY combination for 20 days decreases NC and improves activity QOL indicators without significant side effects or risk for RM. Most Mini RQLQ data are not statistically significant. However, quality of life activity measures are statistically significant with sleep improvement most notable. Relief of nasal congestion trends toward improvement both in nasal congestion scores and QOL scores. It is unclear why most improvement is seen on days 5 to 7. A larger study powered with 145 patients is necessary to prove statistically the effectiveness and safety of these 2 medications used together. There are several papers presented at the American Academy of Allergy Asthma and Immunology, New Orleans, 2010, meeting which demonstrate effectiveness of MNS plus OXY versus one or the other without appreciable side effects [11-13]. It is demonstrated in single-blind control studies that the combination of MNS plus OXY versus either one alone is tolerated and more effective in relieving symptoms of rhinitis. Parameters shown to respond to this combination versus one or the other or placebo include improved onset of action, better relief of nasal congestion, and improved quality of life without any increase in adverse events either during treatment or after such treatment is discontinued [11-13].
